# Throughput Analysis on 3-Dimensional Underwater Acoustic Network with One-Hop Mobile Relay

**DOI:** 10.3390/s18010252

**Published:** 2018-01-16

**Authors:** Xuefeng Zhong, Fangjiong Chen, Jiasheng Fan, Quansheng Guan, Fei Ji, Hua Yu

**Affiliations:** School of Electronics and Information Engineering, South China University of Technology, Guangzhou 510630, China; eexfzhong@mail.scut.edu.cn (X.Z.); fan.js@mail.scut.edu.cn (J.F.); eeqshguan@scut.edu.cn (Q.G.); eefeiji@scut.edu.cn (F.J.); yuhua@scut.edu.cn (H.Y.)

**Keywords:** underwater acoustic communication network, network throughput, mobile relay, dynamic wireless network

## Abstract

Underwater acoustic communication network (UACN) has been considered as an essential infrastructure for ocean exploitation. Performance analysis of UACN is important in underwater acoustic network deployment and management. In this paper, we analyze the network throughput of three-dimensional randomly deployed transmitter–receiver pairs. Due to the long delay of acoustic channels, complicated networking protocols with heavy signaling overhead may not be appropriate. In this paper, we consider only one-hop or two-hop transmission, to save the signaling cost. That is, we assume the transmitter sends the data packet to the receiver by one-hop direct transmission, or by two-hop transmission via mobile relays. We derive the closed-form formulation of packet delivery rate with respect to the transmission delay and the number of transmitter–receiver pairs. The correctness of the derivation results are verified by computer simulations. Our analysis indicates how to obtain a precise tradeoff between the delay constraint and the network capacity.

## 1. Introduction

The theoretical analysis of underwater acoustic communications network (UACN) is of vital importance in various marine applications such as underwater resources exploitation and marine disasters surveillance. Since the network performance is affected by various constraints including link characteristics (e.g., transmission bandwidth and transmission delay), access strategy, scheduling strategy and node distribution in three-dimensional space [1,2], a comprehensive study on network performance taking all limiting factors into account is a substantially difficult task. Existing research has usually considered simplified scenarios. For instance, in [3], the authors studied the performance limits of wireless network capacity and focus on the trade-offs between different parameters and capacity. Most of the studied wireless network scenarios are based on two-dimensional (2D) node deployment. However, underwater acoustic network based on 2D deployment cannot adequately reflect the oceanic phenomena. In fact, it is generally true that realistic underwater sensing networks deployed in three-dimensional (3D) space with parallel transmission of multiple sender–receiver pairs. The method based on 2D wireless network capacity analysis cannot be directly applied to the study of UACN.

Moreover, although considerable research has been devoted to the capacity analysis of UACNs, most existing work only deals with static network [4,5]. Since practical UACNs are usually with sparse node deployment, mobile relay plays an important role in UACN [6]. It is interesting to study the effect of node mobility on network performance, and investigate how to exploit the mobility to improve the network capacity.

In this paper, we consider dynamic UACNs with mobile relays and static communication nodes, and apply the packet delivery rate as the network performance measure. Since underwater acoustic channel has extremely narrow bandwidth and long delay spread, complicated networking protocols (e.g., multi-hop routing) with heavy signaling overhead may not be appropriate. In this paper, we consider only one-hop direct transmission or two-hop transmission via a mobile relay, to save the signaling cost. We derive the closed-form formulation of packet delivery rate with respect to the transmission delay and the number of transmitter–receiver pairs. A systematic analysis of the UACN performance is investigated through rigorous mathematical derivation and simulation. The trade-off relationship between the network throughput and the transmission delay is also presented with simulation and theoretical analysis.

The rest of the paper is organized as follows. First, we discuss related work in Section 2. Then, in Section 3, we describe in detail analysis and simulation results of the one hop transmission. Section 4 provides underwater network throughput analysis based on one-hop mobile relay. Then, the closed-form probability of the underwater acoustic network throughput and simulation results are presented and discussed. Finally, conclusions are given in Section 5.

## 2. Related Work

In wireless network analysis, the capacity of different network models have been studied in [7], where the authors study randomly distributed nodes. The results show that the throughput of nodes decreases gradually with the increase of network nodes, which is due to the fact that each node needs to share the wireless channel with other nodes in its local neighborhood. Grossglauser et al. [8] also demonstrated that it was difficult to achieve high throughput for direct transmission between nodes because most of the time source nodes and target nodes were in a relatively distant position, and the interference caused by parallel transmission has major influence on the network throughput. In order to solve this problem, the authors proposed to relax the transmission delay at the same time allow mobile nodes. It is shown that node mobility improves the network throughput. The throughput of each user is not affected by the increase of nodes.

The work of Grossglauser et al. [8] is the seminal work to explore node mobility. Latterly, Liu et al. [9,10,11,12,13] provide further investigation on the importance of mobile nodes in wireless networks. Liu et al. [9] studied a two-hop network which introduces some transmission delay. In the two-hop network strategy, a number of relay nodes are introduced between the source node and the target node. The packets of the source node need to traverse the relay nodes and transmit the data stream to the destination node via the relay node. The proposed scheme introduces the interference model, relay competition, traffic flow competition and queue delay theory, which are more practical for the capacity analysis in wireless networks. Gamal et al. [11] considered a self-organizing network with a mobile node which can move freely within bounded areas, and the study shows that the throughput of the relay depends on the movement mode of the nodes. The throughput of the configured sink node is significantly better than that of the static wireless network is also proposed in [12]. Li et al. [13] studied how to obtain the smooth tradeoff between the network throughput and the transmission delay by controlling the mobile model of the nodes. The authors allocate *n* nodes which can move in the neighboring cells in several cells. The strategy of multi-hop relay is used to calculate the limit of throughput per node within the permissible delay.

Compared with the fruitful research in terrestrial wireless network, the importance of node mobility is not fully investigated in UACN. The practical topology of underwater acoustic communication network can be statics or dynamic. In the static network structure, the communication nodes which connect the underwater sink nodes are fixed on or anchored to the sea floor. The data of the whole network are conveyed to the surface node through the underwater sink nodes so that the network achieve the full connectivity. The network structure is used for seabed detection and monitoring [14,15]. The dynamic network structure model is based on the static network structure added with mobile relay node [6]. The mobile nodes receiving the data packet of the underwater node sneak to the vicinity of the underwater sink node and deliver the data packet, which effectively reduce the transmission delay and enhance the flexibility of the network with practical significance.

In capacity analysis of underwater acoustic networks, capacity analysis can be divided into link-level capacity analysis and network-level capacity analysis. Stojanovic et al. [16,17,18] provided link-level capacity analysis of point-to-point links. In the theoretical research, Stojanovic et al. [16] derived the relationship between communication bandwidth and distance as well as the traversal capacity for a single link. This model can be extended to an interference link [17], a multiple-input multiple-output link, and a relay-assisted link [18], which are more complex models. Cao et al. [19,20,21] provided the network-level capacity analysis. Stojanovic et al. [19] considered an underwater cellular network with tradeoffs between frequency reuse and maximum feasible user density. They present an analysis of the design of a future ocean observing system for a wide area-covered network architecture based on cellular types. In the underwater acoustic network, the buoy of each surface is equivalent to the base station on the land, which communicates with the subset of nodes of the coverage area. However, since the underwater acoustic channel is narrow band communication, narrow band bandwidth determines that multiple user nodes cannot be accessed at the same time, so the water sound cell network is not commonly used. Stefanov et al. [20] analyzed the performance of water-borne self-organization network in the presence of interference. It is assumed that nodes are distributed uniformly in the deployment area, using channel modeling with path loss and Lace fading. The results show that the network connectivity can be achieved by appropriately selecting the operating frequency, transmission power and bandwidth. The conclusion of the literature can guide us to propose a hierarchical underwater acoustic network architecture, so that there is no cross-interference between the collector network and the sensor network. In [21], a model is proposed to evaluate the network throughput based on the network topology and propagation environment. The nodes are arranged in the network according to the Poisson distribution process. The channel follows the Rayleigh fading distribution, which is easy to obtain the throughput result.

In general, the existing underwater acoustic network capacity analysis is mainly for the static and deterministic network. However, dynamic and random network capacity analysis has not been fully studied. Describing the trade-off relationship between the network capacity and the delay, the transmission strategy and the node mobility model by means of closed-form solution still exhibits significant lack of research attention. The analysis of underwater acoustic capacity is still extremely important research in academia.

## 3. Analysis of One-Hop Transmission

### 3.1. Delivery Rate of One-Hop Transmission

In this section, we shall derive the packet delivery rate of one-hop transmission in a 3D UACN with parallel transmission. Simplified link-layer models are important in the network-level theoretical analysis. There are two commonly applied simplified link-layer models: the physical model and the protocol model [7]. In this paper, we apply the protocol model, which is based on the distance between the sender and the receiver. Successful transmission is obtained when the sender is closer to the receiver than the interfering nodes [7]. Assume all senders have same transmission power and therefore have same effective transmission range *r*. Let $X_{i}$ and $X_{j}$ denote the position of the sender and the receiver, respectively. The transmission is successful if

(1) The distance between $X_{i}$ and $X_{j}$ is not larger than *r*:
  (1) $$\begin{array}{c} |X_{i}-X_{j}|\leq r. \end{array}$$
(2) For all other nodes $X_{k}$ simultaneously transmitting at the same time over the same channel,
  (2) $$\begin{array}{c} |X_{k}-X_{j}|\geq \left(1+\Delta \right)r. \end{array}$$
  where $\left\{X_{k},k\in \Gamma \right\}$ is the subset of source nodes simultaneously transmitting over the same channel. The quantity Δ>0 models situations where a guard zone is specified by the protocol to prevent a neighboring node from transmitting on the same sub-channel at the same time.

Note that node distance is a key parameter in the protocol model. In a random network node, distance is a random variable and it is important to find its probability distribution. In existing work, the distribution of node distance was usually defined as

(3) $$\begin{array}{c} F\left(x\right)=\mathrm{Prob}({\left(x_{s}-x_{r}\right)}^{2}+{\left(y_{s}-y_{r}\right)}^{2}+{\left(z_{s}-z_{r}\right)}^{2}<x^{2}), \end{array}$$

where $\left(x_{s},y_{s},z_{s}\right)$ and $\left(x_{r},y_{r},z_{r}\right)$ denote the locations of the sender and the receiver, respectively. A close-form expression of F(x) has been developed [22,23]. Based on Equation (3), the probabilities in Equations (1) and (2) can be calculated and consequently the theoretical packet delivery rate can be derived.

In underwater communication, it is well accepted that the vertical transmission is more efficient than horizontal transmission. Hence, we argue that ellipsoid-neighborhood should be applied instead of the spherical-neighbor model in underwater acoustic transmission. That is, successful transmission is obtained if the receiver is in a ellipsoid-neighborhood of the sender. In our previous work, we have defined the distribution function of the weighted node distance associated with the ellipsoid-neighborhood, as follows [24]:

(4) $$\begin{array}{c} F\left(x\right)=\mathrm{Prob}\left({\left(x_{s}-x_{r}\right)}^{2}+{\left(y_{s}-y_{r}\right)}^{2}+\frac{1}{\alpha }{\left(z_{s}-z_{r}\right)}^{2}<x^{2}\right),\left(\alpha >1\right), \end{array}$$

where α is the propagation factor which affects the vertical propagation efficiency of underwater acoustic signals. The close-form expression of F(x) has been derived in [24].

**Figure 1:**
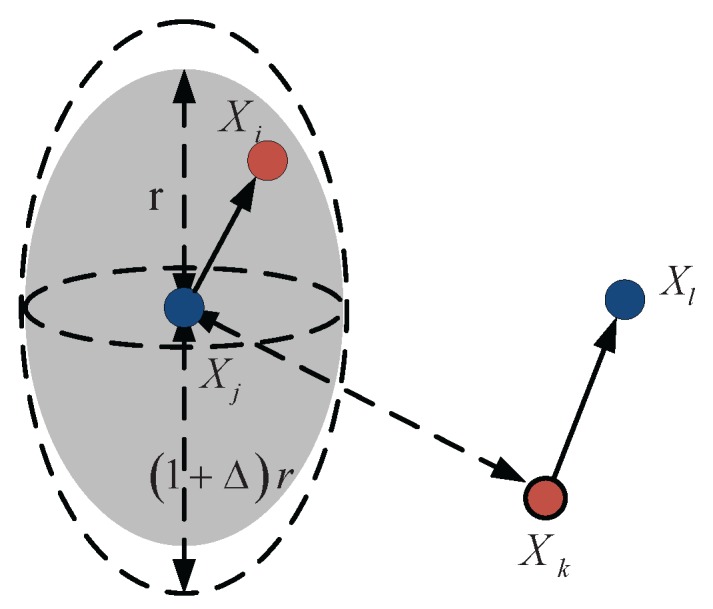
Illusion of the protocol model in a random underwater acoustic communication network.

Based on Equation (4), we now consider the packet delivery rate of one-hop transmission in a 3D UACN. Suppose that the node $X_{j}$ is receiving a packet from the node $X_{i}$ over a underwater acoustic channel. The transmission is successful if there is enough spatial separation from simultaneous transmissions of other nodes, otherwise fails. Let us focus on a time slot. We assume that there are *N* nodes which are randomly deployed in a cuboid area. We fix a sender density parameter ρ∈(0,1), and randomly designate M=ρN senders and receivers, which constitute *M* communication pairs. We randomly select a pair of communication nodes, as shown in Figure 1. When the source node $X_{i}$ transmit packet to the receiver $X_{j}$, the simultaneous transmission of the interference node $X_{k}$ will limit the throughput. The protocol model denotes a range of transmission *r* to avoid the collisions which cause the loss of the packets. Successful transmission is obtained if the source node is in the ellipsoid-neighborhood of the receiver and other interference nodes are out of the ellipsoid-neighborhood of the receiver. For any selected communication node pair, the weighted distance based on (4) is given by

(5) $$\begin{array}{c} d_{ij}=∥X_{i}-X_{j}∥=\sqrt{{\left(x_{s}-x_{r}\right)}^{2}+{\left(y_{s}-y_{r}\right)}^{2}+\frac{1}{\alpha }{\left(z_{s}-z_{r}\right)}^{2}}, \end{array}$$

The probability of successful transmission from node $X_{i}$ to node $X_{j}$ is expressed as

(6) $$\begin{array}{ccc} Prob & = & Prob(d_{ij}\leq r,d_{kj}\geq \left(1+\Delta \right)r),\left(k=1,\cdots ,M,k\neq i\right)\\ & = & Prob\left(d_{ij}\leq r\right)\prod_{k}Prob\left(d_{kj}\geq \left(1+\Delta \right)r\right),\left(k=1,\cdots ,M,k\neq i\right) \end{array}$$

where $d_{kj}$ denotes the weighted distance between interfering nodes and the receiver. In Equation (6), we have assume the nodes are independently deployed.

The probability that the distance of the selected transmission pair is less than the range *r* is actually equivalent to the probability that the distance of any two selected nodes in the network is less than the *r*. We have

(7) $$\begin{array}{c} \begin{array}{c} Prob\left(d_{ij}\leq r\right)=F\left(r\right). \end{array} \end{array}$$

For the *k*th inferencing node, we have

(8) $$\begin{array}{c} \begin{array}{c} Prob\left(d_{kj}\geq \left(1+\Delta \right)r\right)=1-F\left(\left(1+\Delta \right)r\right). \end{array} \end{array}$$

Considering other M−1 transmission pairs, we have

(9) $$\begin{array}{c} \begin{array}{c} Prob\left(d_{kj}\geq \left(1+\Delta \right)r,k=1,\cdots ,M,k\neq i\right)={\left(1-F\left(\left(1+\Delta \right)r\right)\right)}^{M-1}, \end{array} \end{array}$$

where the formula means that other M−1 interference nodes are out of the ellipsoid-neighborhood of the receiver $X_{j}$. In summary, the probability function of successful one-hop transmission in the underwater acoustic network is given by

(10) $$\begin{array}{c} \begin{array}{cc} Prob= & Prob(d_{ij}\leq r,d_{kj}\geq \left(1+\Delta \right)r),k=1,\cdots ,M,k\neq i\\ = & F\left(r\right)\times {\left(1-F\left(\left(1+\Delta \right)r\right)\right)}^{M-1}. \end{array} \end{array}$$

As can be seen from the above formula, the main influence parameters include the distance distribution function of nodes, the propagation factor α, the transmission range *r*, the sender density parameter ρ and the number of nodes *N*. Hence, the packet delivery rate can be defined as Prob(α,r,Δ,ρN).

### 3.2. Optimal Range of One-Hop Transmission

**Figure 2:**
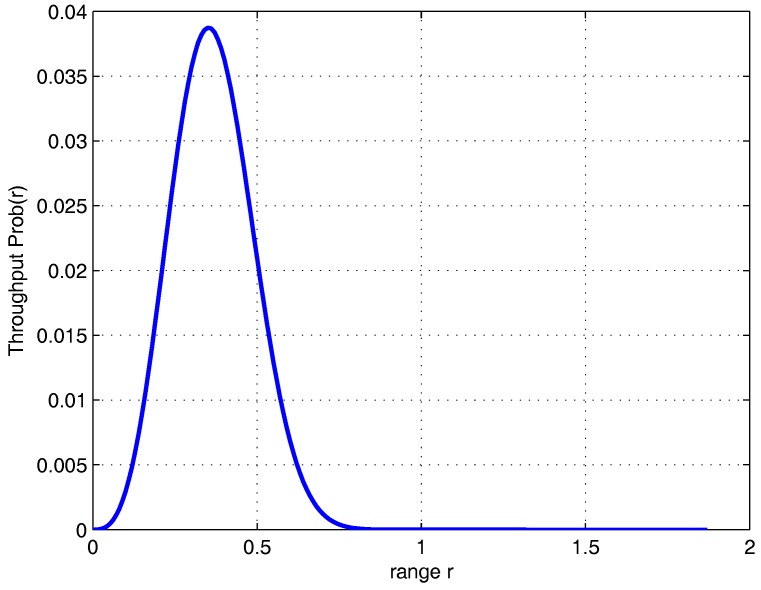
The throughput (in term of packet delivery rate) of one-hop transmission.

In general, the range of the node transmission is determined by the transmission power. When transmission power is too small, the network only achieves local communication. In this case, the network will lose connectivity; however, one can increase the transmission power to achieve large transmission range. However, the mutual interference due to large transmission range will cause collision of packet transmission. Therefore, we should select the appropriate transmission range, both to avoid the loss of packets caused by the transmission collision and preserve the network connectivity. We can set the transmission range as the independent variable and analyze the probability of the one-hop successful transmission. Figure 2 shows the theoretical curve between the throughput and transmission range for N=100,ρ=0.1,α=1.5 and Δ=0.1. It can be observed that Prob(r) is a concave function in its domain. According to the properties of the concave function, any one extreme point of the concave function is the global maximum point on the convex set. Thus, there is an optimal value in its definition domain and the optimal value of the corresponding variable *r* is expressed as

(11) $$\begin{array}{c} r_{opt}=\left\{r|\frac{\partial Prob(r)}{\partial r}=0,r>0\right\}. \end{array}$$

**Figure 3:**
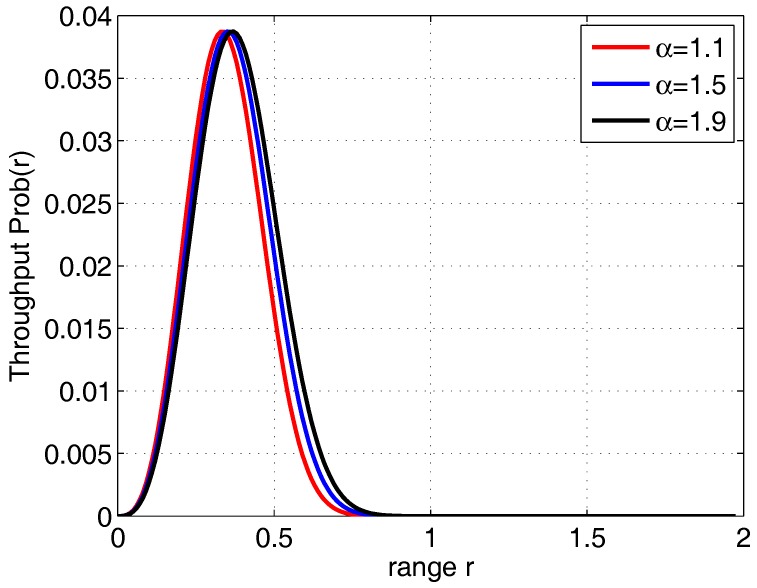
The optimal analysis between the range and throughput.

Then, we change the value of the propagation factor α in the network. As Figure 3 shows, when α changes, the corresponding optimal value of range *r* changes accordingly. The optimal throughput of the one-hop transmission does not change much under the same network parameters.

### 3.3. Simulation of One-Hop Transmission

**Figure 4:**
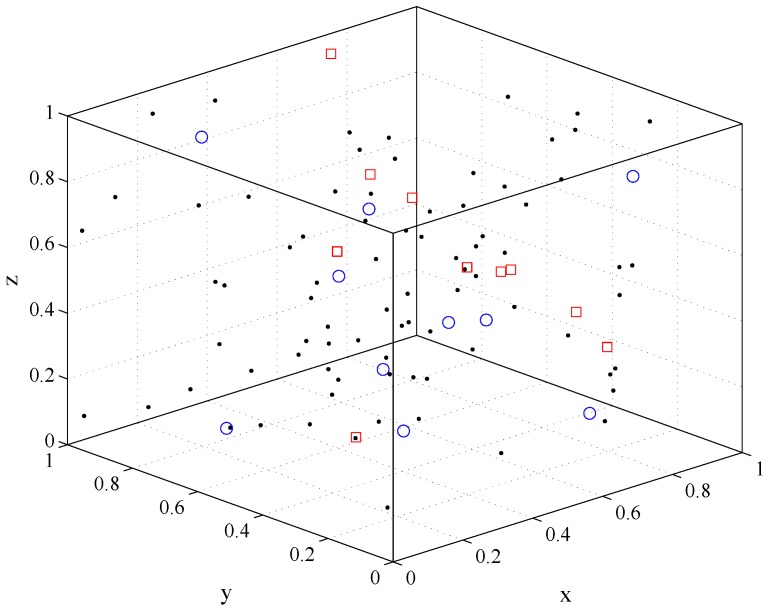
Network node distribution. The red-square denote the source nodes, the blue-circle denote the receivers, and the remaining black nodes are inactive nodes. All axis have normalized range.

Figure 4 shows the simulation scenario. The nodes in the network are uniformly distributed. The red-square nodes are the source nodes, the blue-circle nodes are the receivers, and the remaining black nodes are inactive nodes. In this simulation experiment, we fixed the total number of nodes in the network, to explore the impact of different sender density on the throughput of one-hop transmission. For *X*, *Y*, *Z* axis boundary, we set a=b=c=1. Other parameters are set to α=1.5, N=100 and Δ=0.1. To keep network connectivity, we have chosen the optimal transmission range with different sender density parameters.

**Figure 5:**
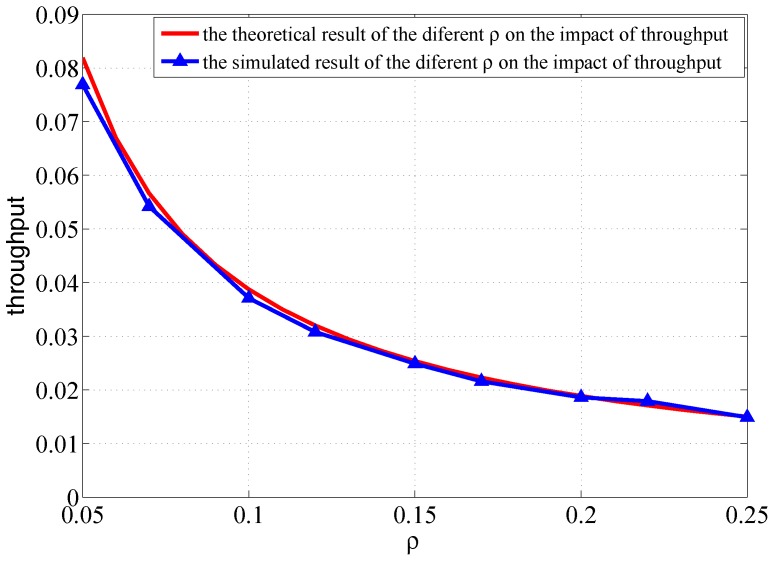
Different sender density on the impact of the throughput of one-hop transmission.

Both the simulated results and the theoretical results are plotted in Figure 5. It can be observed that the throughput of one-hop transmission decreases when the sender density increases. This is because the bottleneck of the node transmission is mainly caused by the collision of the simultaneous transmission. The higher is sender density, the stronger is the interference of simultaneous transmission. The interference between the nodes transmission becomes the main factor that affects the delivery rate of one-hop transmission.

## 4. Transmission Strategy Based on Mobile Relay

In the above section, we derived the closed-form probability of one-hop transmission in the UACN. It can be observed that if only direct transmissions between sources and destinations are allowed, the throughput decreased with the increasing of ρ. This is because the communication pairs are not guaranteed as close as possible, which results in interference caused by simultaneous transmission. If we want to increase throughput beyond this limitation, we should find a transmission strategy to communicate locally as much as possible (to overcome the interference limited). Moreover, we must ensure that there are enough sources and destinations communicating over long distance, which keeps network connectivity (to overcome the coverage limited). Directed communication does not suffice, and we should utilize mobile relay.

### 4.1. The One-Hop Mobile Relay Strategy

Relay nodes are commonly applied in the UACN [6]. Since acoustic signal has a slow propagation speed in water (around 1500 m/s), we argue that multi-hop transmission is not appropriate since it requires complicated signaling, which will lead to unbearable delay. In this paper, we consider one-hop relay. For notation simplicity, we define S→D as direct transmission from the source to the destination, S→R the relay transmission from the source to the relay, and R→D the transmission from the relay to the destination.

**Figure 6:**
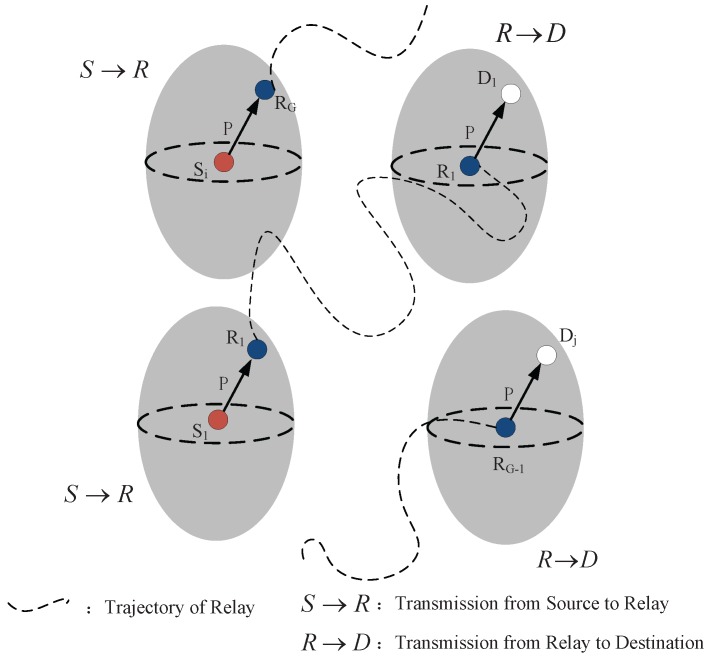
An illustrative example of one-hop relay strategy.

There are *N* fixed nodes uniformly distributed in the network. At the beginning, there are M=ρN nodes requested to transmit packets, and, accordingly, the nodes randomly select other *M* nodes as their receivers. We assumed that every source node transmits one packet in a time slot, so there are *M* packets and communication pairs. We denote them SD pairs. The SD pairs can utilize *G* relays to transmit packets. Once a relay successfully receives the data packet from one source node, it can transfer packet to the destination node via a one-hop transmission until the relay move to position within the transmission range of destination, as shown in Figure 6.

The mobility model of the relays have major influence on the network performance. In existing work on wireless network, the applied mobility models include the i.i.d. (independent and identically distributed) model [25,26,27], the random way-point model [28], the random walk model [29], and the Markovian mobility model [30]. In this paper, we adopt the i.i.d. mobility model, where each mobile relay randomly select a new position independently and identically in each time slot. The i.i.d. mobility model will result in uniformly distributed relay nodes. Hence the analysis of one-hop transmission in Section 3 can be directly applied. We leave other mobility models to future work.

Delay constraint of the transmitted data packet and the buffer size of the relay nodes are also the important limiting factors. In this paper, we focus on the relation between the network throughput and the allowed delay. Hence, we assume all relay nodes have infinite buffer size.

**Figure 7:**
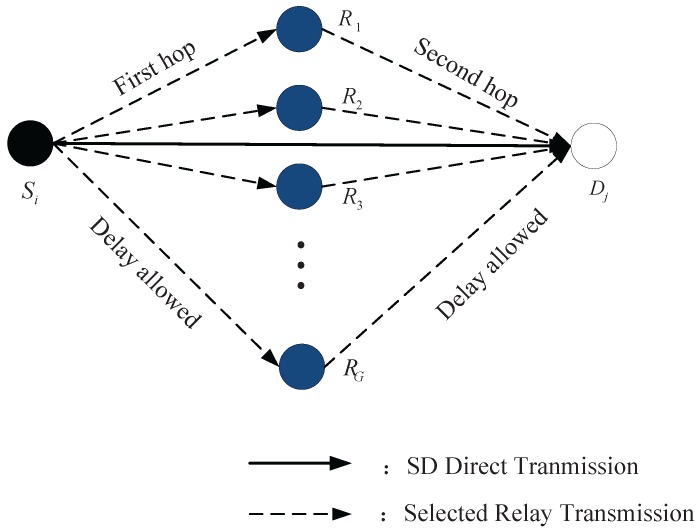
The introduction of two-hop relay strategy.

Note, direct transmission still works if the source node is in the ellipsoid-neighborhood of the receiver and other sources are out of the ellipsoid-neighborhood. We summarize the transmission strategy in Figure 7. There are two modes to deliver the source data packet to the destination nodes. In the first mode, the data packet is transmitted directly from the source node to the destination node. In the second mode, the data packet is delivered from the source node to the relay and then from the relay to the destination node by two hops. All relay nodes can catch packets of multiple source nodes, but one relay just catch or hand off one packet at a time slot due to Autonomous Underwater Vehicle (AUV) works half-duplex in real life. We allow two hop transmission in a time slot by segmenting one time slot into two sub-slots.

### 4.2. Partition of a Time Slot

**Figure 8:**
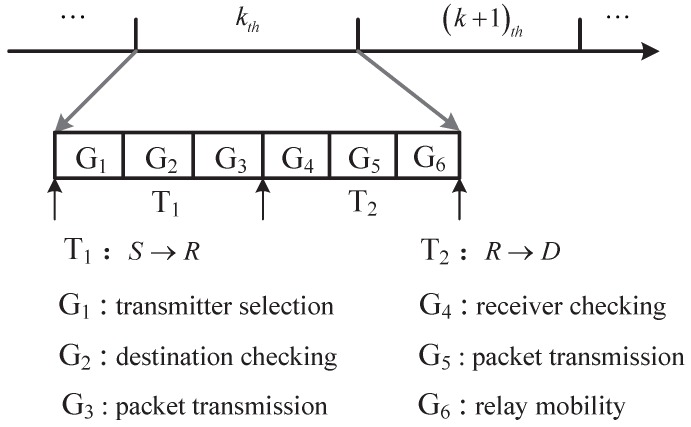
Partition of a time slot.

We consider time-slotted network, which has been adopted as a major medium access technique in UACN [31]. As shown in Figure 8, each time slot is divided into two sub-slots, called sub-slot $T_{1}$ and sub-slot $T_{2}$. In sub-slot $T_{1}$, source data packet is transmitted to the relay or destination. In sub-slot $T_{2}$, the relay node hand off packet from the relay to the destination. We further divide sub-slot $T_{1}$ into three phases. Phase $G_{1}$ is specified for transmitter selection where some nodes are activated to become the transmitter. Furthermore, each transmitter, randomly selects a node as its receiver. In phase $G_{2}$, if the destination node is inside the ellipsoid-neighborhood of the source, packet can be handed off. If not, the source will check whether a idle relay is inside its one hop neighborhood. Phase $G_{3}$ is reserved for packet transmission from source to the selected receiver according to the rule of protocol model. We note that the $G_{1}$ and $G_{2}$ are indeed the hand-shaking periods, which has been widely applied to coordinate the transmission. We also divide $T_{2}$ into three processes. In process $G_{4}$, the relay is checking whether there is a destination inside the ellipsoid-neighborhood of the relay according to the relay buffer data. If so, the relay will transmit packet to it. If not, the relay will stay idle until next slot. In particular, if there are more than one receiver inside the ellipsoid-neighborhood of relay, the relay randomly select one receiver. Process $G_{5}$ is reserved for packet transmission from relay to its destination. The relay will hand off packet to its receiver. Process $G_{6}$ is specified for relay mobility where relay can move according to the relay mobility model. We can get the topology information of the network accurately.

In this paper, we focus on new framework for underwater networking. The above-mentioned new framework includes random network deployment, one-hop relay transmission, new link model and new time-slot structure. The detailed networking protocols, such as the medium access protocol within the time-slot to ensure reliable transmission between source/destination node and relay node, will be left to future work. In the following, we shall propose the analytical tool for the new network framework.

## 5. Theoretical Analysis of Network Throughput Based on Two-Hop Transmission Strategy

In this section, we shall derive the analytical result of the above mentioned two-hop transmission scheme. We assume that, at the beginning, there are *M* senders try to transmit packets to *M* randomly selected receivers.

We shall focus on the tradeoff between throughput and transmission delay. In [8], the throughput is defined as the time-averaged packet delivery rate. In this paper, we apply the similar definition. The applied packet delivery rate is defined as

(12) $$\begin{array}{c} \eta \left(t\right)=\frac{1}{M}E\left[M\left(t\right)\right], \end{array}$$

where *t* denotes the transmission delay and E[M(t)] denotes the mathematical expectation of the packets which are successfully handed off to the destination until the *t*th time slot.

In this section, we shall derive the closed-form expression of the packet delivery rate, and analyze the influence of transmission delay and sender density on the network performance.

### 5.1. Throughput Analysis of One Transmission Pair

We first consider the situation where there is only one communication pair in the network, and there is only one mobile relay. In this case, there is no interference. The probability that the communication pair can transmit successfully is equal to the probability that the receiver is inside the range of one-hop of the source or relay. We denote

- $P_{d}$: The probability that the source packet is directly transmitted to the destination.
- $P_{s}$: The probability that the data packet is successfully transmitted from the sender to the relay.
- $P_{r}$: The probability that the data packet is successfully transmitted from the relay to the receiver.

Based on (4), we have $P_{d}=P_{s}=P_{r}=F\left(r\right)$. Let $P_{T_{1}}\left(t\right)$ denotes the probability that the data packet is successfully caught by the relay node in sub-slot $T_{1}$ of the *t*st time slot. We have

(13) $$\begin{array}{c} \begin{array}{c} P_{T_{1}}\left(1\right)=\left(1-P_{d}\right)\times P_{s}. \end{array} \end{array}$$

Similarly, the probability that the source packet is caught by the relay in the *t*th time slot is

(14) $$\begin{array}{c} \begin{array}{c} P_{T_{1}}\left(t\right)=\left(1-P_{d}\right)\times {\left(1-P_{s}\right)}^{t-1}\times P_{s}. \end{array} \end{array}$$

Next, we consider sub-slot $T_{2}$. Let $P_{T_{2}}\left(t\right)$ denote the probability that the data packet is successfully received by the destination node in time slot *t*. We first consider $P_{T_{2}}\left(1\right)$. It is obvious that the relay can hand off packet to its destination node in the first sub-slot $T_{2}$ when the packet has been caught in the relay and the destination is inside the range of one-hop transmission of the relay node. We have

(15) $$\begin{array}{c} \begin{array}{c} P_{T_{2}}\left(1\right)=P_{T_{1}}\left(1\right)\times P_{r}. \end{array} \end{array}$$

Now, we consider $P_{T_{2}}\left(2\right)$, the probability event includes two cases according to total probability formula. Case 1 is that the packet of source is successfully received by the relay in the first sub-slot $T_{1}$, but the packet is handed off to its destination in the second sub-slot $T_{2}$. Case 2 is that the packet of source is successfully received by the relay in the second sub-slot $T_{1}$, and the packet is handed off to its destination in the second sub-slot $T_{2}$. We have

(16) $$\begin{array}{c} \begin{array}{c} P_{T_{2}}\left(2\right)=P_{T_{1}}\left(1\right)\times \left(1-P_{r}\right)\times P_{r}+P_{T_{1}}\left(2\right)\times P_{r}. \end{array} \end{array}$$

Similarly, if the packet is successfully handed off to destination in the *t*th sub-slot $T_{2}$. The probability event includes *t* cases. Thus, the probability that the packet is successfully handed off to destination in the *t*th time slot is

(17) $$\begin{array}{c} \begin{array}{cc} P_{T_{2}}\left(t\right) & =P_{T_{1}}\left(1\right)\times {\left(1-P_{r}\right)}^{t-1}\times P_{r}\\ & +P_{T_{1}}\left(2\right)\times {\left(1-P_{r}\right)}^{t-2}\times P_{r}\\ & +\cdots +P_{T_{1}}\left(t\right)\times P_{r}. \end{array} \end{array}$$

The probability of successful packet delivery up to the *t*th time slot, denoted as $P_{suc}\left(t\right)$, can be calculated as

(18) $$\begin{array}{c} \begin{array}{c} P_{suc}\left(t\right)=P_{d}+P_{T_{2}}\left(1\right)+\cdots +P_{T_{2}}\left(t\right). \end{array} \end{array}$$

### 5.2. Throughput Analysis of Multiple Transmission Pairs

When studying the throughput of multiple transmission pairs, we have to consider the interference caused by simultaneous transmission. We know that the probability of the successful transmission is Prob(α,r,Δ,M). We set the parameters α,Δ as constants and the one-hop radiation range *r* of the node as the optimal solution under the network parameters. Hence, Prob(α,r,Δ,M) can be simplified to Prob(M).

We first analyze the network throughput in every sub-slot $T_{1}$. Different from the above subsection, we introduce *G* mobile relay into the network. Thus, the probability that each source packet is received by at least one relay is

(19) $$\begin{array}{c} \begin{array}{c} p_{M}=1-{\left(1-Prob\left(M\right)\right)}^{G}. \end{array} \end{array}$$

We temporally ignore the direct transmission. Since there are *M* source nodes, totally, there are *M* packets in the source group. Let $x_{t}$ (t=1,2,⋯) denote in the *t*th time-slot the number of packets that is not successfully transmitted from the source group to the relay nodes. Note that $x_{1}$ is a random variable. Its probability can be presented as

(20) $$\begin{array}{c} \begin{array}{c} \bar{p}\left(x_{1}=m_{1}\right)=C_{M}^{m_{1}}\times p_{M}^{M-m_{1}}\times {\left(1-p_{M}\right)}^{m_{1}}, \end{array} \end{array}$$

where $x_{1}=m_{1}$ denotes that there are $m_{1}$ packets still not being transmitted in the first sub-slot $T_{1}$.

Next, we consider the case that the packets of source group are transmitted to the relay group from the first sub-slot $T_{1}$ to the next sub-slot $T_{1}$. Assuming that, in every slot, a relay can catch only one packet from a source node. The transfer probability that the source group transmit *l* packets is

(21) $$\begin{array}{c} \begin{array}{cc} & \bar{p}\left(x_{2}=m_{2}/x_{1}=m_{1}\right)\\ = & \bar{p}\left(x_{2}=m_{1}-l/x_{1}=m_{1}\right)\\ = & \left\{\begin{array}{cc} C_{m_{1}}^{l}\times p_{m_{1}}^{l}\times {\left(1-p_{m_{1}}\right)}^{m_{1}-l}, & 0\leq l\leq G,m_{1}\geq l\\ 0, & others \end{array}\right., \end{array} \end{array}$$

where $p_{m_{1}}=1-{\left(1-Prob\left(m_{1}\right)\right)}^{G}$ is the probability that the packets of source group are transmitted to the relay group. $x_{2}=m_{2}$ is the packets of source group still not transmitted in the second sub-slot $T_{1}$. *l* is the number of packets that are successfully received by the relay group from the first sub-slot $T_{1}$ to the second sub-slot $T_{1}$, i.e., $m_{2}=m_{1}-l$.

Similarly, we define the transfer probability matrix that the packets of source group are caught by relay group in every sub-slot $T_{1}$. We get

(22) $$P=\left[\begin{array}{cccc} {\bar{p}}_{t}\left(0,0\right) & {\bar{p}}_{t}\left(0,1\right) & \cdots & {\bar{p}}_{t}\left(0,M\right)\\ {\bar{p}}_{t}\left(1,0\right) & {\bar{p}}_{t}\left(1,1\right) & \cdots & {\bar{p}}_{t}\left(1,M\right)\\ \vdots & \vdots & \ddots & \vdots \\ {\bar{p}}_{t}\left(M,0\right) & {\bar{p}}_{t}\left(M,1\right) & \cdots & {\bar{p}}_{t}\left(M,M\right) \end{array}\right],$$

where ${\bar{p}}_{t}\left(i,j\right)=\bar{p}\left(x_{t}=i/x_{t-1}=j\right)$. $x_{t}$ and $x_{t-1}$ are respectively the number of data packets remained in the source group in the current slot and the previous slot. The elements in the transfer probability matrix can be obtained based on Equation (21).

We further define the distribution sequence of the number of source packets at the *t*th time-slot as follows:

(23) $$\begin{array}{c} \begin{array}{c} p_{t}={\left[\begin{array}{cccc} \bar{p}\left(x_{t}=0\right) & \bar{p}\left(x_{t}=1\right) & \cdots & \bar{p}\left(x_{t}=M\right) \end{array}\right]}^{T}. \end{array} \end{array}$$

Considering the direct transmission from source group to destination group, we obtain the initial probability of the number of source packets, as follows:

(24) $$\begin{array}{c} \begin{array}{c} \bar{p}\left(x_{0}=m_{0}\right)=C_{M}^{M-m_{0}}\times {Prob(M)}^{M-m_{0}}\times {\left(1-Prob\left(M\right)\right)}^{m_{0}}. \end{array} \end{array}$$

Based on $p_{0}$, we can determine the distribution sequence of packets remaining in the source group after the first sub-slot $T_{1}$.

(25) $$\begin{array}{c} \begin{array}{c} p_{1}=P\times p_{0}, \end{array} \end{array}$$

where each element in vector $p_{1}$ is given by

(26) $$\begin{array}{c} \begin{array}{cc} \bar{p}\left(x_{1}=m_{1}\right) & =\sum_{m=0}^{M}\bar{p}\left(x_{1}=m_{1}/x_{0}=m\right)\times \bar{p}\left(x_{0}=m\right). \end{array} \end{array}$$

Similarly, we can determine the distribution sequence of the number of data packets remaining in the source group from the second sub-slot $T_{1}$ to the *t*th sub-slot $T_{1}$.

(27) $$\begin{array}{c} \begin{array}{cc} p_{2}= & P\times p_{1}\\ p_{3}= & P\times p_{2}=P^{2}\times p_{1}\\ & \vdots \\ p_{t}= & P^{t-1}\times p_{1} \end{array} \end{array}$$

**Figure 9:**
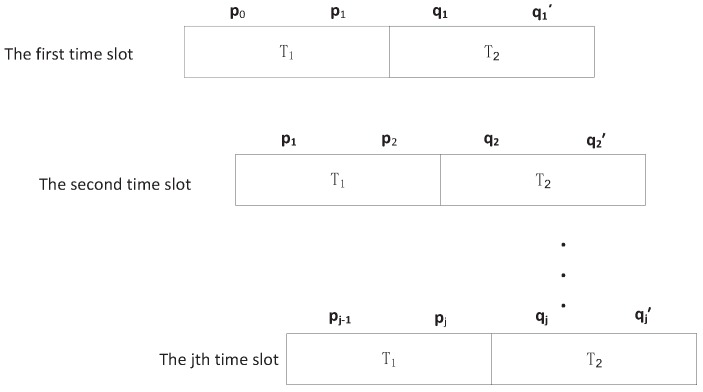
Description of the packet state transition for each time slot.

Next, we consider sub-slot $T_{2}$ in every time slot. As shown in Figure 9, the first step is to determine the distribution of the number of packets caught at the relay group after the first sub-slot $T_{1}$. We define

(28) $$\begin{array}{c} \begin{array}{c} q_{1}={\left[\begin{array}{cccc} \bar{q}\left(y_{1}=0\right) & \bar{q}\left(y_{1}=1\right) & \cdots & \bar{q}\left(y_{1}=M\right) \end{array}\right]}^{T}, \end{array} \end{array}$$

where $\bar{q}\left(y_{1}=n_{1}\right)$ denotes the probability that $n_{1}$ packets are caught at the relay group after the first sub-slot $T_{1}$. Note $q_{1}$ and $p_{1}$ are complementary. The packets transmitted from source group are equivalent to the packets caught at the relay group. Thus, the elements in the vector $q_{1}$ can be obtained by

(29) $$\begin{array}{c} \begin{array}{c} \bar{q}\left(y_{1}=n_{1}\right)=C_{m_{0}}^{n_{1}}\times p_{m_{0}}^{n_{1}}\times {\left(1-p_{m_{0}}\right)}^{m_{0}-n_{1}}. \end{array} \end{array}$$

The second step is to determine the distribution sequence of packets left at the relay group after the first sub-slot $T_{2}$. Before the first sub-slot $T_{2}$ we assume that there are $y_{1}=n_{1}$ packets caught at the relay group and the packets are uniformly distributed in the relay group. Thus, each relay node has an average of $\frac{n_{1}}{G}$ packets. Since each relay only transmit one packets in a slot, there is at most one packets being received by the destination node for a relay. The packets left in the relay group need to be transmitted in the next slot. Thus, the probability that the packets of relay group are handed off to destination is

(30) $$\begin{array}{c} \begin{array}{c} q_{n_{1}}=1-{\left(1-P_{r}\right)}^{\frac{n_{1}}{G}}, \end{array} \end{array}$$

where $P_{r}=Prob\left(G\right)$. Note, in sub-slot $T_{2}$, only the relay nodes can transmit data. The transfer probability can be calculated by assuming the relay group transmit $\bar{l}$ packets, as follows:

(31) $$\begin{array}{c} \begin{array}{cc} & \bar{q}\left(\bar{y_{1}}=\bar{n_{1}}/y_{1}=n_{1}\right)\\ = & \bar{q}\left(\bar{y_{1}}=n_{1}-\bar{l}/y_{1}=n_{1}\right)\\ = & \left\{\begin{array}{cc} C_{G}^{\bar{l}}\times q_{n_{1}}^{\bar{l}}\times {\left(1-q_{n_{1}}\right)}^{G-\bar{l}}, & 0\leq \bar{l}\leq G,\bar{l}\leq n_{1}\\ 0, & others \end{array}\right., \end{array} \end{array}$$

where $\bar{y_{1}}=\bar{n_{1}}$ denotes the number of packets left at the relay group after the first sub-slot $T_{2}$. Thus, we can define the transfer probability matrix Q for every sub-slot $T_{2}$.

(32) $$Q=\left[\begin{array}{cccc} {\bar{q}}_{t}\left(0,0\right) & {\bar{q}}_{t}\left(0,1\right) & \cdots & {\bar{q}}_{t}\left(0,M\right)\\ {\bar{q}}_{t}\left(1,0\right) & {\bar{q}}_{t}\left(1,1\right) & \cdots & {\bar{q}}_{t}\left(1,M\right)\\ \vdots & \vdots & \ddots & \vdots \\ {\bar{q}}_{t}\left(M,0\right) & {\bar{q}}_{t}\left(M,1\right) & \cdots & {\bar{q}}_{t}\left(M,M\right) \end{array}\right],$$

where ${\bar{q}}_{t}\left(i,j\right)=\bar{q}\left(\bar{y_{t}}=i/y_{t}=j\right)$. The elements in the transfer probability matrix can be obtained by Equation (31). Thus, the distribution of the number of packets caught at the relay group $q_{1}$ after the first sub-slot $T_{2}$ is defined as

(33) $$\begin{array}{c} \begin{array}{c} q_{1}^{\prime }={\left[\begin{array}{cccc} {\bar{q}}^{\prime }\left(\bar{y_{1}}=0\right) & {\bar{q}}^{{}^{\prime }}\left(\bar{y_{1}}=1\right) & \cdots & {\bar{q}}^{{}^{\prime }}\left(\bar{y_{1}}=M\right). \end{array}\right]}^{T} \end{array} \end{array}$$

We get

(34) $$\begin{array}{c} \begin{array}{c} q_{1}^{\prime }=Q\times q_{1}, \end{array} \end{array}$$

where the elements in the vector $q_{1}^{{}^{\prime }}$ can be obtained by

(35) $$\begin{array}{c} \begin{array}{cc} {\bar{q}}^{{}^{\prime }}\left(\bar{y_{1}}=\bar{n_{1}}\right) & =\sum_{n_{1}=0}^{M}\bar{q}\left(\bar{y_{1}}=\bar{n_{1}}/y_{1}=n_{1}\right)\times \bar{q}\left(y_{1}=n_{1}\right). \end{array} \end{array}$$

Then, we should determine $q_{2}$ through $p_{1},q_{1}^{{}^{\prime }}$, as shown in Figure 9. Let $n_{2}$ denote the number of packets in the relay group after the 2nd sub-slot $T_{1}$. Note $\bar{n_{1}}$ packets of the relay group increase to $n_{2}$ because some packet are transmitted from the source group to the relays. We should calculate how many packets of source group are successfully transmitted to the relay group given $p_{1}$. In other words, there are $\bar{m}=n_{2}-\bar{n_{1}}$ packets transmitted from the source group into the relay group after the process $p_{1}\to p_{2}$. The transfer probability is given by

(36) $$\begin{array}{c} \begin{array}{cc} & \tilde{p}\left(z_{1}=\bar{m}/x_{1}=m_{1}\right)\\ = & \left\{\begin{array}{cc} C_{m_{1}}^{\bar{m}}\times p_{m_{1}}^{\bar{m}}\times {\left(1-p_{m_{1}}\right)}^{m_{1}-\bar{m}}, & \bar{m}\leq m_{1}\\ 0, & others \end{array}\right., \end{array} \end{array}$$

where $z_{1}=\bar{m}/x_{1}=m_{1}$ denotes that the source group transmit $z_{1}=\bar{m}$ packets successfully to the relay group under the condition that the source group has $x_{1}=m_{1}$ packets. Thus, we can determine the transfer probability that there are $n_{2}$ packets caught in the relay group after the second sub-slot $T_{1}$ under the condition that there are $m_{1}$ packets in the source group and $\bar{n_{1}}$ packets in the relay group. We have

(37) $$\begin{array}{c} \begin{array}{cc} & \bar{q}\left(y_{2}=n_{2}/x_{1}=m_{1},\bar{y_{1}}=\bar{n_{1}}\right)\\ = & \tilde{p}\left(z_{1}=\bar{m}/x_{1}=m_{1}\right). \end{array} \end{array}$$

Thus, we can define the transfer probability matrix $\tilde{P}$ as

(38) $$\tilde{P}=\left[\begin{array}{cccc} {\tilde{p}}_{t}\left(0,0\right) & {\tilde{p}}_{t}\left(0,1\right) & \cdots & {\tilde{p}}_{t}\left(0,M\right)\\ {\tilde{p}}_{t}\left(1,0\right) & {\tilde{p}}_{t}\left(1,1\right) & \cdots & {\tilde{p}}_{t}\left(1,M\right)\\ \vdots & \vdots & \ddots & \vdots \\ {\tilde{p}}_{t}\left(M,0\right) & {\tilde{p}}_{t}\left(M,1\right) & \cdots & {\tilde{p}}_{t}\left(M,M\right) \end{array}\right],$$

where ${\tilde{p}}_{t}\left(i,j\right)=\tilde{p}\left(z_{t}=i/x_{t}=j\right)$. The elements in the transfer probability matrix can be obtained by (37). We get

(39) $$\begin{array}{c} \begin{array}{c} q_{2}=\tilde{P}\times q_{1}^{{}^{\prime }}, \end{array} \end{array}$$

where the elements in the vector $q_{2}$ is given by

(40) $$\begin{array}{c} \begin{array}{cc} & \bar{q}\left(y_{2}=n_{2}\right)\\ = & \sum_{\bar{n_{1}}=0}^{M}\bar{q}\left(y_{2}=n_{2}/x_{1}=m_{1},\bar{y_{1}}=\bar{n_{1}}\right)\cdot {\bar{q}}^{{}^{\prime }}\left(\bar{y_{1}}=\bar{n_{1}}\right). \end{array} \end{array}$$

Similarly, we can determine the distribution sequence of packets remaining at the relay group from the second sub-slot $T_{2}$ to the *t*th sub-slot $T_{2}$. We have

(41) $$\begin{array}{c} \begin{array}{cc} q_{2}= & \tilde{P}\times q_{1}^{{}^{\prime }}\\ q_{2}^{{}^{\prime }}= & Q\times q_{2}\\ q_{3}= & \tilde{P}\times q_{2}^{{}^{\prime }}\\ & \vdots \\ q_{t-1}^{{}^{\prime }}= & Q\times q_{t-1}\\ q_{t}= & \tilde{P}\times q_{t-1}^{{}^{\prime }}\\ q_{t}^{{}^{\prime }}= & Q\times q_{t}. \end{array} \end{array}$$

Note that $p_{t}$ and $q_{t}^{{}^{\prime }}$ indicate how many data packets remain in the source group and relay group, respectively. Based on $p_{t}$ and $q_{t}^{{}^{\prime }}$, we can determine how many packets have been successfully delivered to the destination nodes up to the *t*th time slot. Firstly, we calculate the mathematical expectation of the packets remaining in the source group and the relay group, as follows:

(42) $$\begin{array}{c} \begin{array}{c} E\left[x_{t}\right]=\sum_{m=0}^{M}m\times \bar{p}\left(x_{t}=m\right), \end{array} \end{array}$$

(43) $$\begin{array}{c} \begin{array}{c} E\left[\bar{y_{t}}\right]=\sum_{\bar{n}=0}^{M}\bar{n}\times {\bar{q}}^{{}^{\prime }}\left(\bar{y_{t}}=\bar{n}\right). \end{array} \end{array}$$

Then, the mathematical expectation of the packets which are successfully handed off to the destinations, up to the *t*th time slot, is given by

(44) $$\begin{array}{c} \begin{array}{c} E\left[M\left(t\right)\right]=M-E\left[x_{t}\right]-E\left[\bar{y_{t}}\right], \end{array} \end{array}$$

and consequently the throughput can be calculated based on Equation (12).

## 6. Simulation of Network Throughput Based on Two-Hop Transmission Strategy

### 6.1. Simulation of One Transmission Pair

**Figure 10:**
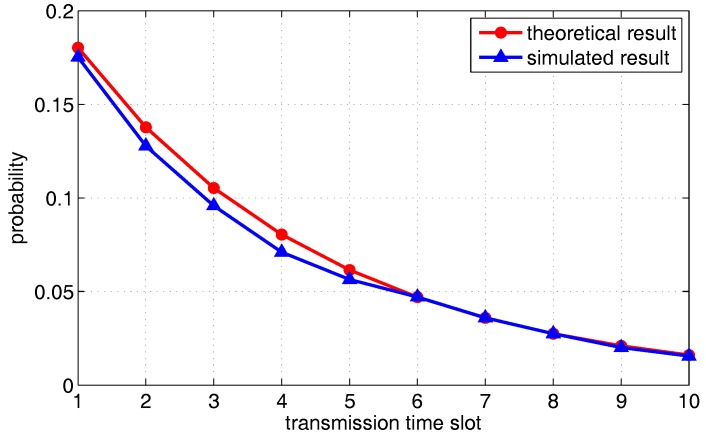
The probability of the source packets be transmitted to relays, as a function of transmission delay.

**Figure 11:**
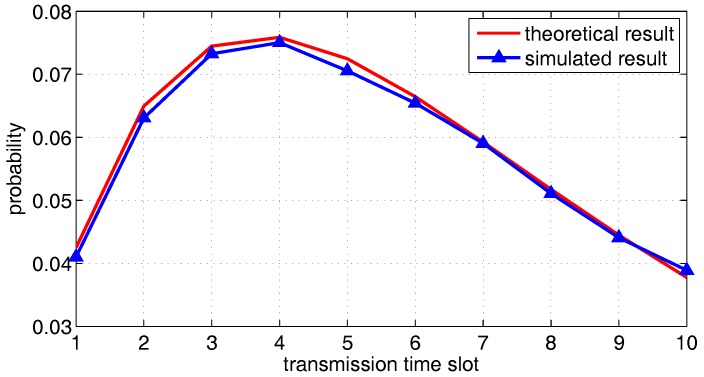
The probability of the packet in relay group be transmitted to destination nodes, as a function of transmission delay.

We first compare the theoretical results of one transmission pair derived in the previous section against the outcome of simulations. For *X*, *Y*, *Z* axis boundary, we set a=b=c=1. Other parameters are set to α=1.5, N=2, Δ=0.1, r=0.5 and the number of mobile relay points G=1. Figure 10 shows the probability (i.e., $P_{T_{1}}\left(t\right)$ in Equation (14)) that the source packets are transmitted to the relay nodes in different transmission slots. Figure 11 shows the probability that a packet of the relay is successfully handed off to the destination (i.e., $P_{T_{2}}$ in Equation (17)). It can observed that the theoretical result is generally consistent with the simulated result, which verifies the correctness of the derived theoretical result.

It can be observed in Figure 11 that $P_{T_{2}}$ reaches a peak value after some delay. The proposed scheme is more suitable for delay tolerant service. Nevertheless, a large delay is not required. Figure 11 shows that, after 10 time slots, more than 70% of the source packets will be delivered to the destination nodes.

### 6.2. Simulation of Multiple Transmission Pairs

**Figure 12:**
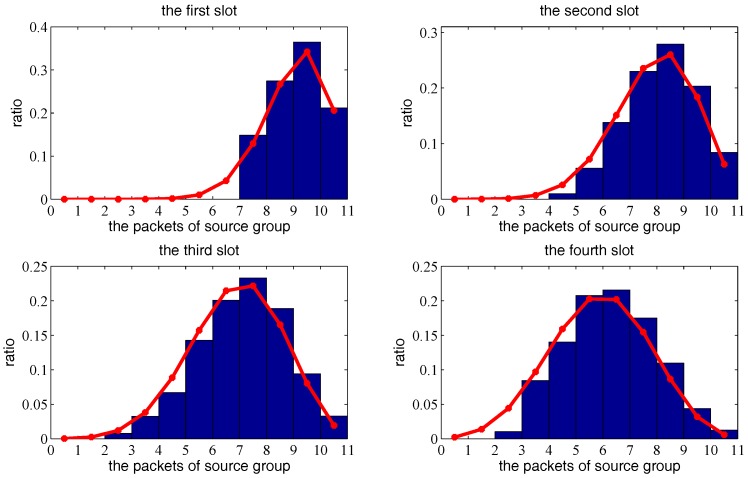
The distribution sequence of the source group based on two-hop mobile relay transmission strategy from the first slot to the fourth slot, as a function of the transmission delay. The red curve is the theoretical result. The blue histogram is the simulated result.

**Figure 13:**
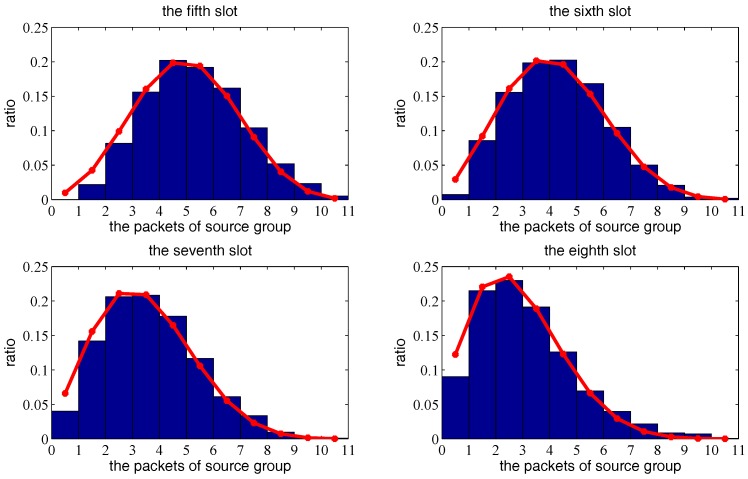
The distribution sequence of the source group based on two-hop mobile relay transmission strategy from the fifth slot to the eighth slot, as a function of the transmission delay. The red curve is the theoretical result. The blue histogram is the simulated result.

**Figure 14:**
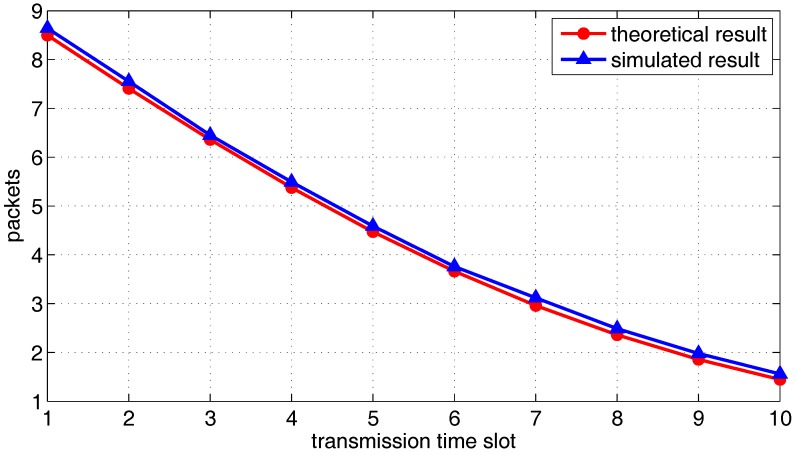
The mathematical expectation of the number of source packets be transmitted to the relay group, from the first slot to the tenth slot.

Next, we consider multiple transmission pairs based on two-hop transmission. The *X*, *Y*, *Z* axis boundary is set to a=b=c=1, and other parameters are set to α=1.5, N=100,ρ=0.1, G=3, r=0.35 and Δ=0.1. Figure 12 and Figure 13 show the theoretical probabilities distribution of packets left at source group and the simulated histogram under 1000 independent trials, from the first time-slot to the eighth time slot. Figure 14 shows the mathematical expectation of packet numbers in different transmission slots. There is very good agreement between the analytical model and simulated results, which verifies the correctness of the previous derivation.

**Figure 15:**
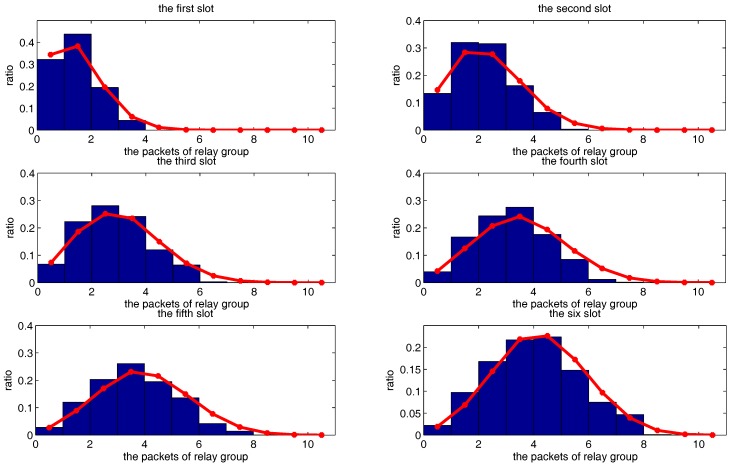
The distribution sequence of the packets left at the relay group, from the first slot to the sixth slot. The red curve is the theoretical result. The blue histogram is the simulated result.

**Figure 16:**
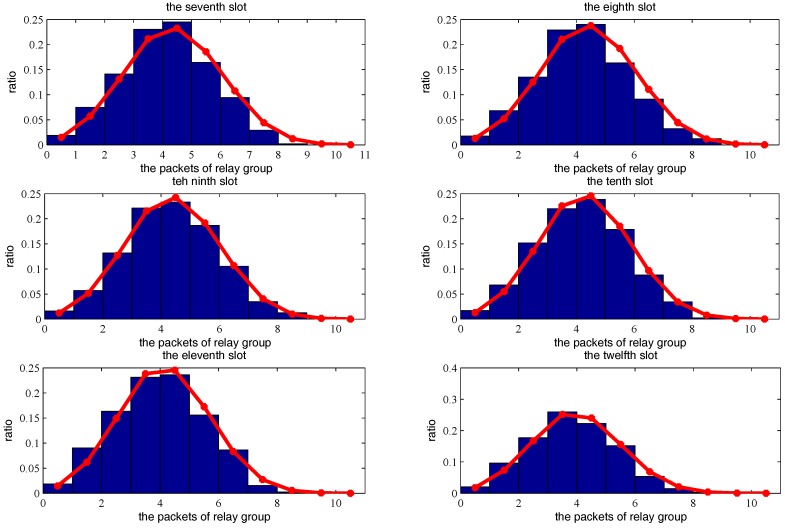
The distribution sequence of the packets left at the relay group, from the seventh slot to the twelfth slot. The red curve is the theoretical result. The blue histogram is the simulated result.

**Figure 17:**
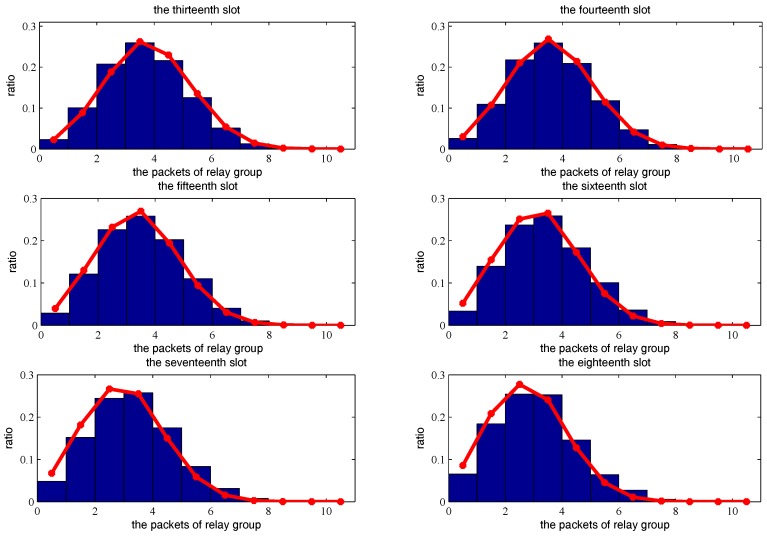
The distribution sequence of the packets left at the relay group, from the thirteenth slot to the eighteenth slot. The red curve is the theoretical result. The blue histogram is the simulated result.

**Figure 18:**
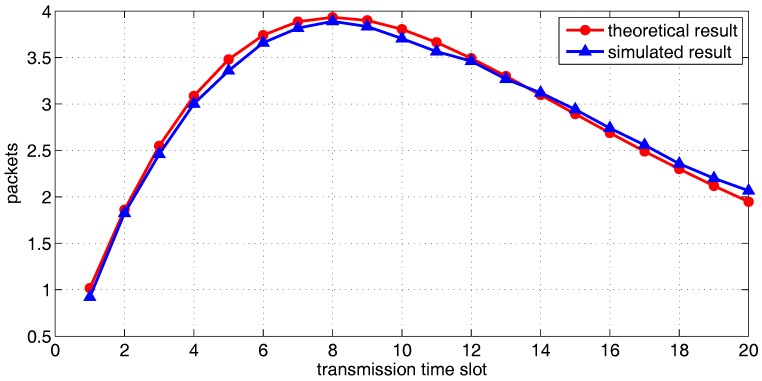
The mathematical expectation of the number of data packets left in the relay group, from the first slot to the twentieth slot.

Figure 15, Figure 16 and Figure 17 show the theoretical and simulated distribution sequence of packets left at the relay group, from the first time-slot to the eighteenth time-slot. Figure 18 shows the mathematical expectation of packet numbers in different transmission slots. Good agreement between the analytical model and simulated results also can be observed. The packets of relay group reach the peak in the eighth time slot but begins to decline gradually in the later time slots.

Comparing Figure 11 with Figure 18, one can find that for multiple communication pairs, it takes much larger delay to deliver the source packets. This is because multiple communication pairs lead to mutual interference and therefore lower the probability of successful one-hop transmission. One can introduce more mobile relays to reduce the delay.

**Figure 19:**
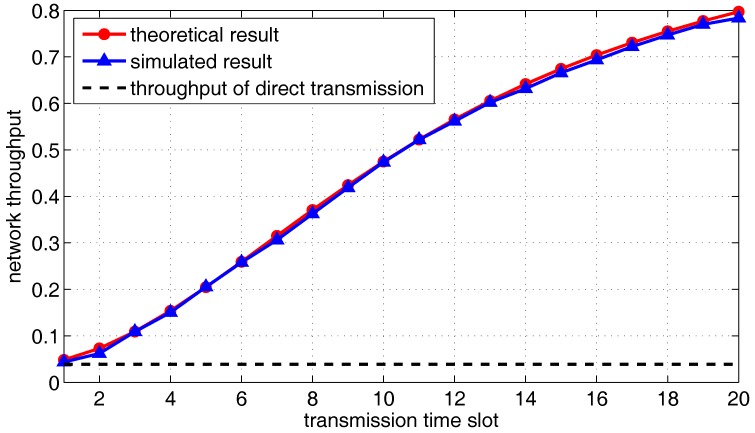
The overall delivery rate as a function of the transmission time slots.

Finally, the overall packet delivery rate η(t) is presented in Figure 19, where both theoretical results and simulated results are plotted. The result of direct transmission is also plotted for comparison. As shown in Figure 19, the network throughput increases over time and is much better than the case where only direct transmission is allowed. The gradient of throughput reaches the maximum in the eleventh slot but slowly decreases in the later time slot. Therefore, corresponding to different parameters of UACN model, we can set the optimal transmission delay according to the theoretical derivation, which achieves high network throughput based on appropriate delay.

## 7. Conclusions

In this paper, we have investigate a new framework for UACN. The new framework has the following features: (1) randomly deployed source nodes and destination nodes; (2) mobile relay with i.i.d. mobility model; (3) one-hop relay transmission; and (4) new simplified link model and slot structure. Moreover, we also derived the theoretical packet delivery rate of the proposed framework. We first derive the throughput based on one communication pair and then extend the result to multiple communication pairs. The network throughput analysis derivation process is validated through simulations.

Regarding further research, one could study the closed-form formulation of one-hop transfer under the underwater acoustic network physical model. Additionally, the mobility model of the relay nodes is of vital importance. More practical mobility models, such as the Random Way-point Mobility Model, should be investigated based on the proposed framework. It is also interesting to develop practical networking protocols based on the proposed framework, such as medium access protocol within the time slot.
